# Severe Neonatal Alloimmune Thrombocytopenia in a Multiparous Female With No Prior History

**DOI:** 10.7759/cureus.28181

**Published:** 2022-08-19

**Authors:** Trenton Judd, Martha P Tomsic

**Affiliations:** 1 Pediatrics, Liberty University College of Osteopathic Medicine, Lynchburg, USA; 2 Internal Medicine-Pediatrics, Baptist Memorial Union County, New Albany, USA

**Keywords:** term neonate, intracranial haemorrhages, neonatal alloimmune thrombocytopenia, petechiae, severe thrombocytopenia

## Abstract

Neonatal Alloimmune Thrombocytopenia (NAIT) is the most common cause of severe thrombocytopenia in newborns. It is also the most common cause of morbidity and mortality in full-term infants that present with severe thrombocytopenia, given its association with intracranial hemorrhage (ICH). NAIT can present in many ways depending on the severity of platelet destruction. The patient's presentation can range from asymptomatic or can include more serious symptoms such as petechial rash and ICH. Due to potentially fatal outcomes of undiagnosed severe NAIT, it is imperative that patients are identified, diagnosed, and treated in a timely and efficient manner. We report a case of NAIT in a newborn male infant who initially was asymptomatic and eventually developed a petechial rash that encompassed the torso and groin as the only signs of disease. Given the importance of the timely diagnosis and treatment of NAIT and its potentially fatal outcomes, the aim of this case report is to help clinicians recognize the presentation of NAIT and the steps in treating it.

## Introduction

Neonatal Alloimmune Thrombocytopenia (NAIT) is a rare form of thrombocytopenia in newborns and is estimated to be reported in one in 1,200 births, for mild presentations of the disease, and up to one in 2,000 births for severe presentations every year [1]. NAIT is caused by maternal antibodies that are formed against an alloantigen that is found on fetal platelets [2], specifically linked to the human platelet antigen (HPA) 1a and 5b [3]. NAIT is categorized as mild (platelet count of 100,000-150,000/μL), moderate (platelet count of 50,000-99,000/μL), or severe (platelet count of <50,000/μL) according to the definition of the disease on UpToDate [4]. Some of the clinical manifestations are cephalohematoma, petechiae, purpura, and gastrointestinal hemorrhage, with severe cases leading to intracranial hemorrhage (ICH) and even death if untreated [5,6]. Treatments that are currently available and have been shown to be effective are steroids given after birth in mild cases with platelet transfusions of platelets that are HPA-1a and 5b negative and intravenous immune globulin (IVIG) used in cases of severe disease or in cases of persistent hemorrhage [7]. If there is a history of maternal immune thrombocytopenia (ITP), splenectomy, or previous pregnancy that resulted in NAIT, precautions, such as a complete blood count (CBC) at the time of birth to rule out thrombocytopenia, should be taken at the time of delivery to provide quick testing and diagnosis of a newborn with NAIT so treatment will not be delayed [8,9].

## Case presentation

**Figure 1:**
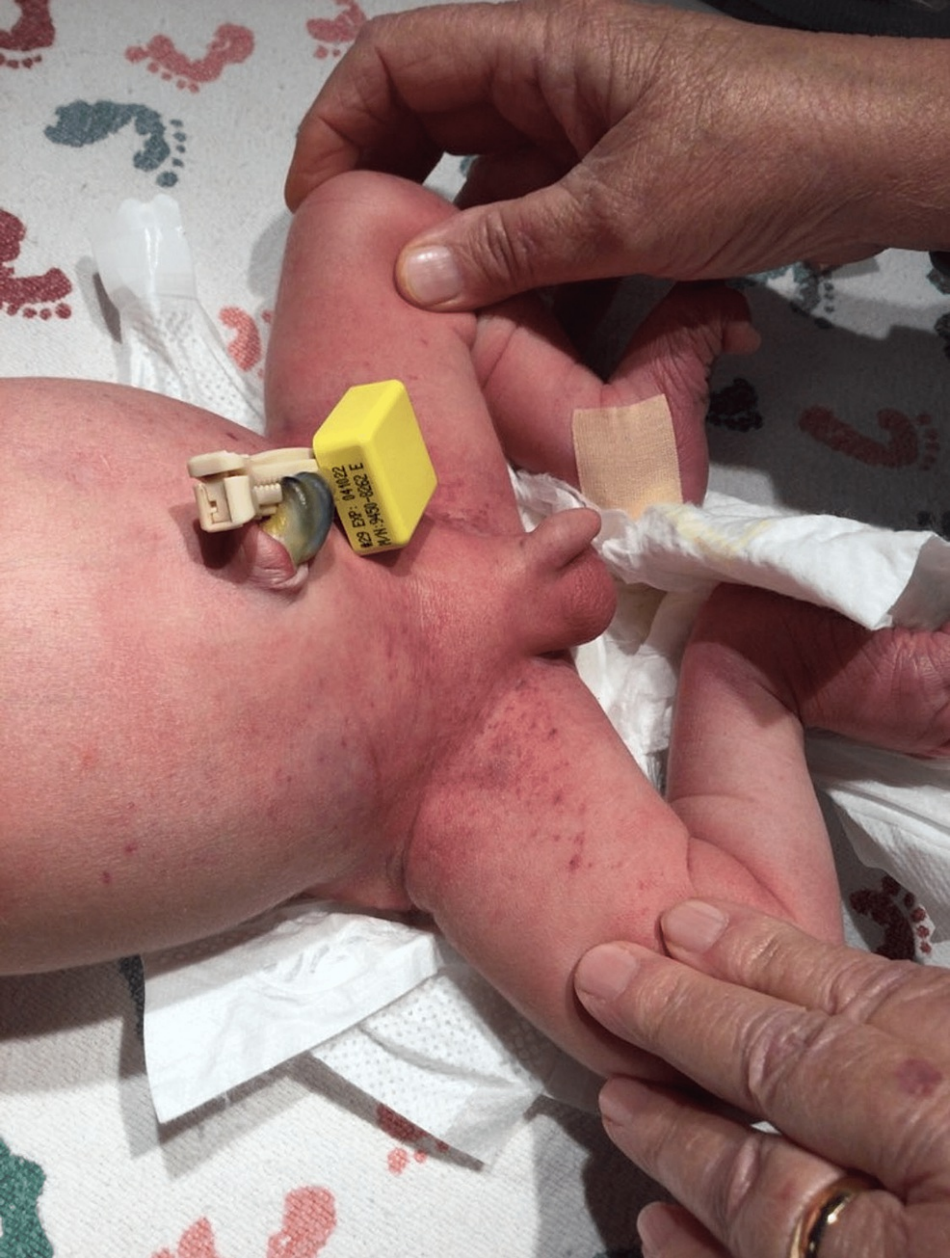
Initial petechial rash in the groin and perineal area

The patient was a term newborn male delivered vaginally at 39 weeks and three days of gestation to a gravida 7, now para 6 mother without any complications. The patient’s mother received routine and adequate prenatal care throughout her pregnancy. The mother does not have a history of immune thrombocytopenia, which could have been a risk factor in this pregnancy [8]. There were no anomalies noted on prenatal scans and prenatal labs were all normal. The patient transitioned well after delivery and obtained APGAR (Appearance, Pulse, Grimace, Activity, and Respiration) scores of 7 and 9 at one and five minutes, respectively. Several hours after delivery it was noted by the nursing staff that the patient had a small petechial eruption in the perineal and groin region that was covered by his diaper (Figure 1).

**Figure 2:**
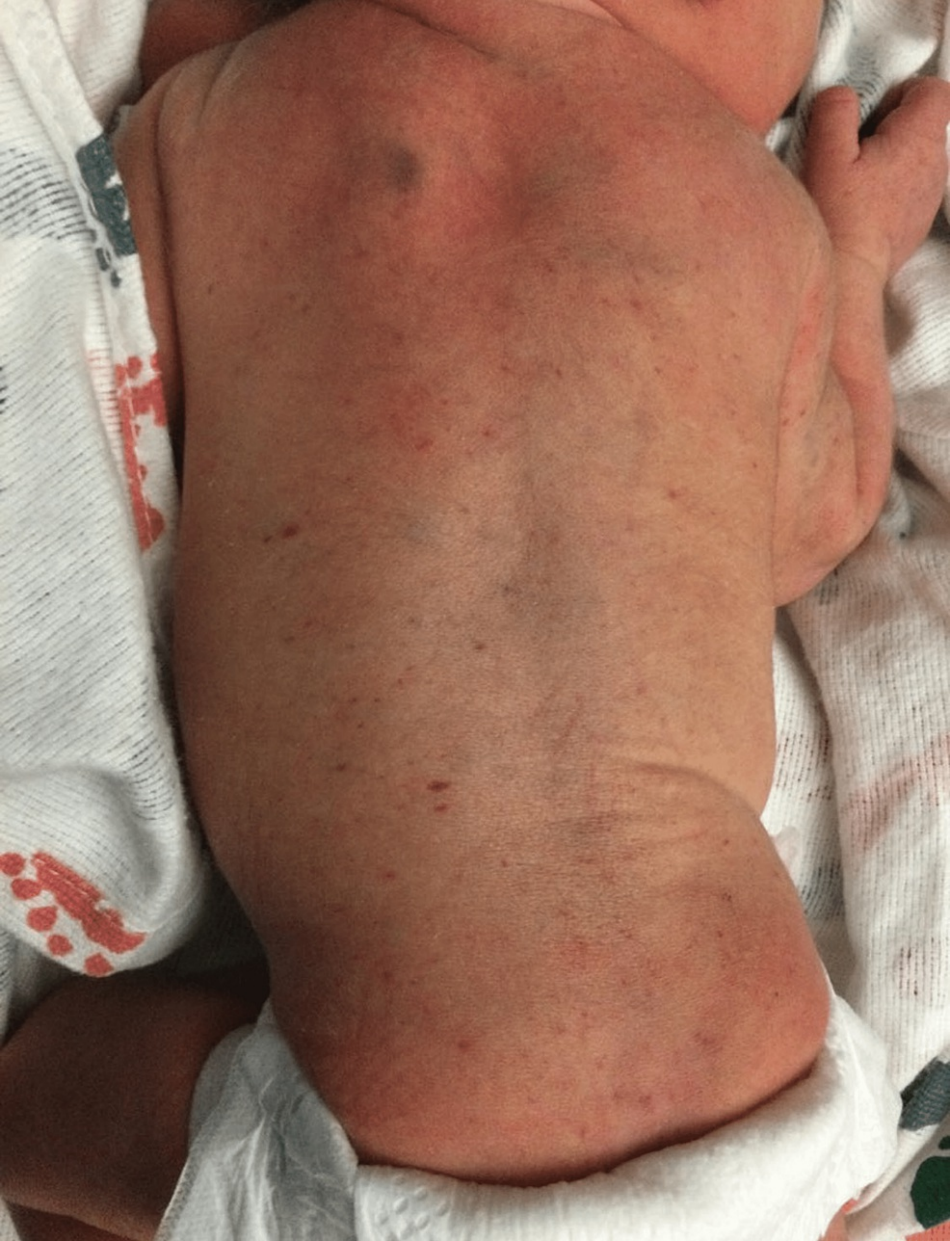
The spread of the petechial rash to the back that presented 16 hours after delivery

The patient was monitored for several hours and between 16 and 18 hours of life, the petechial rash spread to include the torso (Figure 2). At this time, a complete blood count (CBC) and coagulation testing were performed (Table 1).

His vitals signs were as follows: blood pressure (BP) of 66/31 mmHg, a pulse of 150 beats per minute, body temperature of 98.2 ^o^F, respiratory rate of 46 breaths per minute, saturation of peripheral oxygen (SpO2) of 94% on room air, a length of 19 inches (20^th^ percentile), a weight of 6 pounds 8.8 ounces (20^th^ percentile), and a body mass index (BMI) of 12.75kg/m^2^. His lab findings are reported in Table 1 [10]. 

**Table 1:** Lab values of the patient compared to normal reference ranges

Variables	Patient value	Normal range
Hemoglobin (Hb)	18.1 g/dL	11.0-17.3 g/dL
Hematocrit (Hct)	54.3%	35.4-56.5%
White blood cell (WBC)	22.9 K/uL	3.1-21.6 K/uL
Platelets (Plt)	32 K/uL	152-472 K/uL

The laboratory values from the CBC demonstrated that the patient had severe thrombocytopenia. Due to the risk of bleeding and ICH the patient was then transferred to a hospital that could provide a higher level of care and treatment. The patient was evaluated by ultrasound of the head which is the screening method of choice for ICH, and it was determined that he did not have signs of hemorrhage. He was also tested for congenital infections to rule out an infectious cause of thrombocytopenia. Due to the negative test results and otherwise healthy appearance and absence of malformation, the patient was diagnosed with severe NAIT. In order to reduce the risk of ICH the patient was started on a platelet transfusion. This improved the patient’s platelets to 43,000 with coagulation studies remaining normal. The patient was then admitted to the neonatal intensive care unit (NICU) and subsequently treated with intravenous immune globulin (IVIG) was administered in 1g/kg doses every 24 hours. The patient received two doses in the hospital before discharge. The patient responded well to the platelet infusions and IVIG therapy and was subsequently discharged after a five-day hospital stay for further monitoring. 

## Discussion

NAIT is caused by the opsonization of fetal platelets by maternal antibodies. Many cases are considered to be mild and newborns in these cases need little to no treatment [1,6]. According to UpToDate, severe cases, defined as a platelet count of <50,000, are estimated to make up 10% of NAIT cases overall [4]. Although there are some maternal factors that can help predict which newborns might be at risk for NAIT, such as maternal immune thrombocytopenic purpura (ITP) or a previous pregnancy that resulted in NAIT, screening is not performed for mothers who have not exhibited any of these symptoms. It is estimated that 50% of cases that are reported occur in the first pregnancy and because of this, maternal symptoms or risk factors will not be identified. If a case of severe NAIT is left undiagnosed or unidentified, it is estimated that 10-20% of these cases will result in ICH with resulting congenital hydrocephalus [11].

With the increased risk of ICH in severe NAIT, recognition of signs and symptoms is followed promptly by treatment with a platelet infusion that is HPA-1a and 5b negative, steroids, IVIG, or a combination of these three treatments. Treatment for severe NAIT usually begins with platelet transfusion of HPA-1a and 5b negative plasma the first-line treatment of choice and is then escalated to IVIG therapy where available [1,8]. Treatment is aimed at limiting the risk of negative outcomes such as hydrocephalus, gastrointestinal hemorrhage, or even death. Newborns that are categorized as having severe NAIT should be screened with cranial ultrasound to rule out ICH which is the most common cause of death. Newborns should continue to be monitored by physical appearance and serial platelet counts over a period of 48-72 hours or greater to ensure that these serious outcomes are prevented. When the platelet count begins to rise, and there have been no signs of serious side effects, newborns may be discharged home with their caregiver or guardians with regular follow-up provided.

Regarding this patient, there were no maternal risk factors identified prior to birth. The mother had five previous uncomplicated deliveries prior to the patient. Within hours post-delivery, the infant was identified by the attending physician and hospital staff to have a petechial rash that encompassed the groin and perineal area. The patient was carefully monitored and 16-18 hours post-delivery the petechial rash was noted to have spread to include both the anterior and posterior torso. A CBC was ordered that showed severe thrombocytopenia with platelets of 32,000 with rule out testing of other congenital and infectious causes of significant thrombocytopenia negative, such as cytomegalovirus (CMV), toxoplasma, rubella, or HIV [9]. Recognizing the level of care the patient would need, as well as possible platelet infusions and IVIG, the decision was made to transfer the patient to a tertiary hospital in a neighboring city. The patient was given the correct treatment and was screened for ICH using cranial ultrasound. The patient responded well to treatment and after a four-day hospital stay showing improvement in his platelet counts and no new symptoms, was able to be discharged home with his mother and father the next day. The mother should be monitored in future pregnancies and precautions should be taken at the time of delivery to ensure any subsequent infant is screened and treated accordingly.

## Conclusions

NAIT is an uncommon disease among newborns but is the leading cause of severe thrombocytopenia and ICH in the newborn population. As a CBC is not a routine part of newborn care, clinicians should have a high index of suspicion if there are signs of thrombocytopenia in a newborn infant, such as petechiae, purpura, or abnormal bleeding. Subsequently, if there is a history of maternal ITP or previous delivery that resulted in NAIT, newborns should be screened with a CBC as the prompt diagnosis and treatment of NAIT is necessary to lessen the likelihood of death or life-long sequelae related to this condition. These newborns should also be screened for ICH using a cranial ultrasound within 24 hours for the risk of ICH and life-long sequala or even death is incredibly high in these infants.

## References

[1] Curtis BR (2015). Recent progress in understanding the pathogenesis of fetal and neonatal alloimmune thrombocytopenia. Br J Haematol.

[2] Peterson JA, McFarland JG, Curtis BR, Aster RH (2013). Neonatal alloimmune thrombocytopenia: pathogenesis, diagnosis and management. Br J Haematol.

[3] Zdravic D, Yougbare I, Vadasz B (2016). Fetal and neonatal alloimmune thrombocytopenia. Semin Fetal Neonatal Med.

[4] Marion DW (2022). Neonatal thrombocytopenia: clinical manifestations, evaluation, and management. https://www.uptodate.com/contents/neonatal-thrombocytopenia-clinical-manifestations-evaluation-and-management#!.

[5] (2022). Fetal and neonatal alloimmune thrombocytopenia- about the disease. (2021). Accessed: July 20. https://rarediseases.info.nih.gov/diseases/2295/fetal-and-neonatal-alloimmune-thrombocytopenia.

[6] Espinoza JP, Caradeux J, Norwitz ER, Illanes SE (2013). Fetal and neonatal alloimmune thrombocytopenia. Rev Obstet Gynecol.

[7] te Pas AB, Lopriore E, van den Akker ES, Oepkes D, Kanhai HH, Brand A, Walther FJ (2007). Postnatal management of fetal and neonatal alloimmune thrombocytopenia: the role of matched platelet transfusion and IVIG. Eur J Pediatr.

[8] Point F, Terriou L, Rakza T, Drumez E, Alluin G, Garabedian C, Houfflin-Debarge V (2022). Risk factors for severe neonatal thrombocytopenia in cases of maternal immune thrombocytopenia. Acta Paediatr.

[9] Roberts I, Murray NA (2008). Neonatal thrombocytopenia. Semin Fetal Neonatal Med.

[10] Al-Marzoki JM, Al-Maaroof ZW, Kadhum AH (2012). Determination of reference ranges for full blood count parameters in neonatal cord plasma in Hilla, Babil, Iraq. J Blood Med.

[11] Póvoa AM, Ramalho C, Machado AP, Matias A, Montenegro N (2007). Congenital posthemorrhagic hydrocephalus: a case of fetomaternal alloimmune thrombocytopenia. Fetal Diagn Ther.

